# Influence of Age of Infection on the Gut Microbiota in Worker Honey Bees (*Apis mellifera iberiensis*) Experimentally Infected with *Nosema ceranae*

**DOI:** 10.3390/microorganisms12040635

**Published:** 2024-03-22

**Authors:** Daniel Aguado-López, Almudena Urbieta Magro, Mariano Higes, Juan Miguel Rodríguez, Raquel Martín-Hernández

**Affiliations:** 1 Laboratorio de Patología Apícola, Centro de Investigación Apícola y Agroambiental (CIAPA), Instituto Regional de Investigación y Desarrollo Agroalimentario y Forestal (IRIAF), Consejería de Agricultura de la Junta de Comunidades de Castilla-La Mancha, Camino de San Martín s/n, 19180 Marchamalo, Spain; almudena.urbieta@gmail.com (A.U.M.); mhiges@jccm.es (M.H.); 2 Departamento de Nutrición y Ciencia de los Alimentos, Facultad de Veterinaria, Universidad Complutense de Madrid (UCM), 28040 Madrid, Spain; jmrodrig@ucm.es

**Keywords:** microbiota, *Nosema*, honey bees, age of infection, qPCR, *Gilliamella apicola*

## Abstract

The gut microbiota of honey bees has received increasing interest in the past decades due to its crucial role in their health, and can be disrupted by pathogen infection. *Nosema ceranae* is an intracellular parasite that affects the epithelial cells of the midgut, altering gut homeostasis and representing a major threat to honey bees. Previous studies indicated that younger worker bees are more susceptible to experimental infection by this parasite, although the impact of infection and of age on the gut bacterial communities remains unclear. To address this, honey bees were experimentally infected with a consistent number of *N. ceranae* spores at various ages post-emergence (p.e.) and the gut bacteria 7 days post-infection (p.i.) were analysed using real-time quantitative PCR, with the results compared to non-infected controls. Infected bees had a significantly higher proportion and load of *Gilliamella apicola*. In respect to the age of infection, the bees infected just after emergence had elevated loads of *G. apicola*, *Bifidobacterium asteroides*, *Bombilactobacillus* spp., *Lactobacillus* spp., *Bartonella apis*, and *Bombella apis*. Moreover, the *G. apicola* load was higher in bees infected at nearly all ages, whereas older non-infected bees had higher loads of *Bifidobacterium asteroides*, *Bombilactobacillus* spp., *Lactobacillus* spp., *Ba. apis*, and *Bo apis*. These findings suggest that *N. ceranae* infection and, in particular, the age of bees at infection modulate the gut bacterial community, with *G. apicola* being the most severely affected species.

## 1. Introduction

Pluricellular organisms have been exposed to microorganisms constantly throughout their evolution, resulting in close co-evolutionary relationships between an organism’s microbiota and the host. Consequently, diverse microbial communities are established in different host tissues, with the gastrointestinal tract being the site of highest microbial density and diversity [1]. The honey bee microbiota has received increasing attention in recent years, with studies attempting to address the mutualistic and pathogenic associations to better understand the intrinsic relationships with bee health [2,3,4,5]. Gut bacterial communities are less diverse in adult bees and these populations are very specialized, establishing stable, co-dependent symbiotic relationships that seem to be crucial for the animal’s health and well-being. Furthermore, there is little variation between individuals within the same colony or between colonies, seasons, and geographical regions [6].

In the honey bee gut microbiota, eight bacterial taxa account for 95–99.9% of the *16S rRNA* gene sequences in the gut [7,8,9]. Among them, five taxa appear to be found in all bees and are considered to be the “core gut microbiome”, including *Gilliamella apicola*, *Snodgrassella alvi*, *Bifidobacterium asteroides*, *Bombilactobacillus* spp. (previously Firm-4), and *Lactobacillus* spp. (previously Firm-5). The other three taxa are found in all colonies, but not in all bees: *Frischella perrara*, *Bartonella apis*, and *Bombella apis*. All of these bacteria are distributed differentially throughout the bee gut, such that *Bo. apis* can be found in the crop, and it is usually found in pollen, nectar, and hive materials [10,11]. Together with *Ba. apis*, this species is also found in the midgut [12]. As for the other bacteria, *F. perrara* is found in the pylorus [13], *G. apicola* and *S. alvi* in the ileum forming a biofilm [14,15], and *B. asteroides*, *Bombilactobacillus* spp., and *Lactobacillus* spp. in the rectum [16,17].

Through metamorphosis, honey bees pass through different developmental stages: larva, pupa, and adult. In the first stage, bacterial exchange may occur during the feeding of larvae [18], although the gut lining is then shed during pupation, resulting in almost complete elimination of any attached gut bacteria [19]. Hence, once metamorphosis is complete, the new adult bee lacks gut bacteria, yet when it gnaws through the operculum, it begins to acquire residual bacteria from the operculum and through contact with other hive elements and/or older bees [20,21]. Indeed, it is estimated that the characteristic gut microbiota of the bees is established by 7 days post-emergence (p.e.) and it remains constant in terms of bacterial diversity [14,22].

The honey bee microbiota plays many crucial roles in the host, promoting food digestion [23,24,25], enhancing host development [26], modulating behaviour [27], detoxifying pesticides [28], protecting against microplastics [29], enhancing the immune response, and protecting against parasites and pathogens [30,31,32]. Among the various pathogens infecting honey bees, *Nosema ceranae* is one of the most widely distributed worldwide [33,34,35,36,37,38]. This microsporidium is an obligate intracellular sporulating parasite that infects midgut cells, using the host’s machinery to obtain the resources it needs to proliferate and ultimately destroy the epithelial cells of this tissue [39,40]. Microsporidium infection alters the bee’s vital functions, causing energetic stress [41], disturbing immune responses [40,42,43], and altering olfaction, learning, orientation, and memory [44], as well as provoking digestive disorders. Overall, these problems lead to accelerated bee ageing [45] and they interfere with the tasks the bees must perform in the hive [46], ultimately augmenting mortality at the individual and colony levels [47].

*N. ceranae* mainly infects adult bees, although it has also been observed at other developmental stages [48,49]. Indeed, susceptibility to microsporidium infection appears to be influenced by the age of the bees. Field studies indicate that bees inside the colony can first be infected 4–5 days p.e., depending on the season [50], and that older worker bees have higher parasite loads [50,51,52]. However, both younger queens [53] and younger workers [54] are more susceptible to *N. ceranae* infection than older ones in experimental laboratory infections. These differences may be influenced by the gut microbiota [54], although there has been little research into this issue to date. In fact, *N. ceranae* infection can modify the relative abundance of some bacterial species [55,56,57,58] since altering the gut epithelium may modify how the gut microbiota is established. Therefore, the aim of this study was to shed light on this issue and to determine whether *N. ceranae* infection itself, and the age at which bees are infected, following a standard method for microsporidia infection, influences their gut bacterial communities.

## 2. Materials and Methods

### 2.1. Sample Selection

The samples used in this study were a subset of the *Apis mellifera iberiensis* bees used in an earlier study [54] aimed at determining how the age of infection by *N. ceranae* affects the parasite load. In that study, potential colony-level influences were mitigated by obtaining capped brood frames from 5 healthy *Nosema*-free colonies (as confirmed by PCR testing). The frames were kept at 35 °C in an incubator to ensure a continuous supply of newly emerged *Nosema*-free honey bees of a known age. All of the newly emerged workers were carefully extracted from the brood combs each day and randomly placed in steel mesh cages until infection. In this way, all of the bees in each cage were of the same age. The bees were fed ad libitum with a freshly prepared sucrose solution (50% *w*/*w* in dH_2_O) supplemented with 2% Promotor L^®^ (Calier Lab., Les Franqueses del Vallès, Spain), a commercial mixture of amino acids and vitamins.

The bees were anaesthetised with CO_2_ and when they started to wake up, they were infected (on day 0, 1, 4, 5, 8, 11, 13, or 14 p.e.) by administering 2 µL of a spore solution (57,000 spores/µL) purified on Percoll^®^ 95% [59] from naturally infected bees into the mouthparts. The spore solution was vortexed after every third bee to ensure that the suspension remained uniform, and control bees of the same age were fed individually with 2 µL of spore-free water. At 7 days post-infection (p.i.), the number of surviving bees was recorded and they were sacrificed for molecular analysis. A total of 276 bee abdomens were analysed: infected (N = 215) and non-infected (N = 61) bees. The age and number of bees used to analyse the gut microbiota are shown in Table 1. It was confirmed that all bees in the infected group were positive for *N. ceranae* infection and those in the control group were not infected [54].

### 2.2. Molecular Analysis

The abdomen of each bee was carefully separated from the thorax under sterile conditions to avoid contamination between samples, and DNA extraction was performed individually in a final volume of 100 µL as described previously [54]. In addition, the spore solution used as an inoculum to infect the bees was analysed to ensure it was free of gut bacteria.

The absolute abundance (bacterial load) of the main honey bee gut bacteria (*F. perrara*, *G. apicola*, *S. alvi*, *B. asteroides*, *Bombilactobacillus* spp., *Lactobacillus* spp., and *Ba. Apis*) was determined by real-time quantitative PCR (qPCR), targeting the *16S rRNA* gene with previously described primers [23] and normalised to the host (*Apis mellifera*) cytochrome oxidase I (*Am*-*COI*) [60].

In addition, a primer pair and a probe were designed to detect and quantify *Bo. apis* (previously Alpha 2.2). The *16S rRNA* gene sequences of *Bo. apis*, as well as *Parasaccharibacter apium* and *Saccharibacter floricola* that were both recently reclassified as *Bo. apis* [61], were obtained from GenBank (NCBI; Table S1) and aligned using ClustalW Multiple Alignment software (BioEdit Sequence Alignment Editor 7.2.6.1). A conserved fragment of 151 bp present in all of the available sequences was selected and analysed with Primer Express 3.0.1 software (Applied Biosystems Life Technologies Corp.; Foster City, CA, USA). For primer and probe design, the best primer pair and probe were selected based on the parameters determined by the software (stability, length, and % G/C), and they were provided by Roche Diagnostics (Basel, Switzerland). The probe chosen was modified by adding locked nucleic acids (LNAs) to increase the binding temperature to match that of the primers (Table 2).

All samples were tested individually and in duplicate in 384-well plates using a LightCycler^®^480 thermal cycler (Roche Diagnostics, Basel, Switzerland). Negative controls were also tested in parallel for all qPCRs. Bacterial and *Am*-*COI* qPCR reactions were carried out in a final volume of 10 µL LightCycler^®^ 480 SYBR Green I Master mix (Roche Diagnostics, Basel, Switzerland), with each primer at 0.3 µM and 1 µL of the DNA template. The qPCR conditions involved: an initial denaturation of 5 min at 95 °C; 45 cycles at 95 °C for 10 s, 60 °C for 10 s and 72 °C for 10 s; and a final cooling step of 30 s at 40 °C; a melting curve at 95 °C for 5 s; 1 min at 65 °C followed by cooling at 4 °C; and a final 30 s cooling step at 40 °C, to check that the amplicons obtained were of the expected size. 

For *Bo. apis* qPCR, reactions were carried out duplicate in a final volume of 10 µL containing 3.2 µL H_2_O (Sigma Aldrich, St. Louis, MO, USA), 5 µL LightCycler^®^ 480 Probes Master mix (Roche Diagnostics, Basel, Switzerland), 0.3 µM each primer, 0.1 µM probe, and 1 µL DNA template. The PCR conditions involved an initial denaturation of 10 min at 95 °C; 45 cycles at 95 °C for 10 s, 60 °C for 30 s, and 72 °C for 1 s. The amplification cycle of each sample was analysed with the LightCycler^®^ 480 c1.5.1 software (Roche Diagnostics) using the Second Derivate Maximum statistical algorithm to calculate the Crossing Point (Cp).

To quantify the bacterial load, synthetic double-stranded oligonucleotides (gBlock^®^ Gene Fragment, IDT, DNA Technologies; Coralville, IA, USA) were designed from the reference sequences of the target gene for each of the bacterial species tested (Table S2). The synthetic DNAs were reconstituted according to the manufacturer’s instructions and they were used to elaborate standard curves based on serial dilutions with known amounts of synthetic DNA encoding the target sequence. The copy number of the synthetic DNA was calculated from its molecular weight and the DNA concentration in the solution. Dilutions containing between 2 and 10^8^ copies of synthetic DNA per µL were used to generate the standard curves. The limit of detection (LOD) for each bacterium was set according to the last Cp value (lowest DNA concentration) that generated an amplification signal. Thus, for *F. perrara*, *G. apicola*, and *S. alvi*, the LOD was set at 10^2^ copies of DNA; for *B. asteroides*, *Bombilactobacillus* spp., *Lactobacillus* spp., and *Ba. apis*, 10 copies were used as the LOD; and the LOD for *Bo. apis* was set at 2 copies. Bacterial targets for which the DNA copies were below the LOD were considered to be too low to be quantified and, therefore, they were considered negative and quantified as 0 copies. Primer efficiency (E) was estimated from the slope of the equation, E = 10^(−1/slope)^, and the characteristics of the primers and their performance are summarised in Table S3. Subsequently, the bacterial loads were normalised to the *Am*-*COI* gene copy number, and assessed with dilutions of synthetic *Am-COI* DNA contained between 10^2^ and 10^8^ copies. Samples for which the honey bee *COI* value was negative or less than 10^5^ copies were excluded from the analysis as the DNA extracted was considered to be of poor quality.

Normalisation to the *Am*-*COI* gene was performed for all samples to reduce the variation in gut size and DNA extraction efficiency. The copy number of the *16S rRNA* gene of each bacterium was determined as described previously [23]. First, the raw copy number (n_raw_) of each target was calculated in 1 µL of DNA (the volume used in each qPCR reaction) based on the Cp automatically extrapolated to the standard curve using the thermal cycler software mentioned above. The raw copy number was then normalised by dividing it by the number of *Am*-*COI* gene copies present in the sample (n*_COI_*), which was determined in the same way. This normalised *16S rRNA* gene copy value was then multiplied by the median *Am*-*COI* gene copy number of all samples and the total volume of the DNA extracted (i.e., 100 µL) to obtain the normalised copy numbers per abdomen (n_abs_): n_abs_ = (n_raw_/n*_COI_*) × median (n*_COI_*) × 100. Once the data were normalised, they were converted to a logarithmic scale for statistical analysis and all the values considered as 0 copies were replaced by 1 for the logarithmic transformations.

### 2.3. Statistical Analysis

To determine whether *N. ceranae* infection had any effect on the presence of any bacterial group, a chi-squared test with Yates correction was performed for each of the bacterial species analysed. In addition, in *Nosema*-infected and non-infected bees, the bacterial load of each species tested was compared using the Games–Howell (GH) post hoc test. The bacterial load of infected and non-infected bees was compared in each age cohort using the non-parametric Mann–Whitney (MW) test to assess whether the age of infection with *N. ceranae* produced differences in the load of any bacterial group. All analyses were carried out using IBM^®^ SPSS^®^ Statistics 25.0 software, considering values of *p* < 0.05 as statistically significant.

## 3. Results

### 3.1. Influence of N. ceranae Infection on the Honey Bee Gut Bacteria

In order to determine whether the species of gut bacteria and their load differed in infected and non-infected bees, 13 samples (9 infected and 4 non-infected bees) out of the 276 bee abdomens available were excluded from the analysis due to the lack of amplification or because they were below the LOD for *Am*-*COI* established. The spore solution used to infect the bees was negative for all of the bacterial species assessed in this study, indicating that the infected bees were not inoculated with gut bacteria.

**Figure 1:**
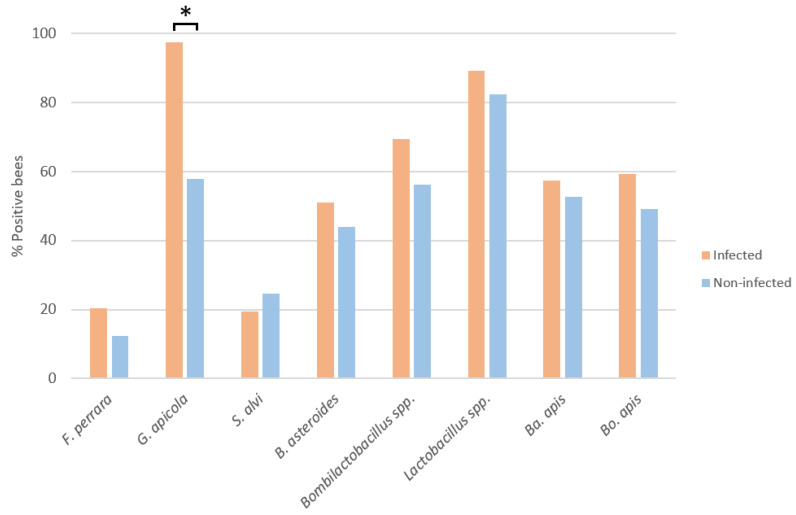
Percentage (%) of bees positive for each gut bacterial species in infected and non-infected bees, irrespective of age. * Statistically significant difference (*p* < 0.05).

The proportions of infected and non-infected bees positive for each bacterium analysed were established (Figure 1). In the infected bees, there was an increase in the presence of almost all bacterial species relative to the non-infected bees. However, after the chi-squared test, only *G. apicola* was in a significantly higher proportion of infected bees (X^2^ = 67,658; *p* < 0.0001). The proportions with the remaining bacterial species did not differ significantly between the groups: *F. perrara* (X^2^ = 1.44; *p* = 0.230), *S. alvi* (X^2^ = 0.13; *p* = 0.716), *B. asteroides* (X^2^ = 0.641; *p* = 0.423), *Bombilactobacillus* spp. (X^2^ = 2.964; *p* = 0.085), *Lactobacillus* spp. (X^2^ = 1.38; *p* = 0.240), *Ba. apis* (X^2^ = 0.226; *p* = 0.634), and *Bo. apis* (X^2^ = 1.469; *p* = 0.225).

**Figure 2:**
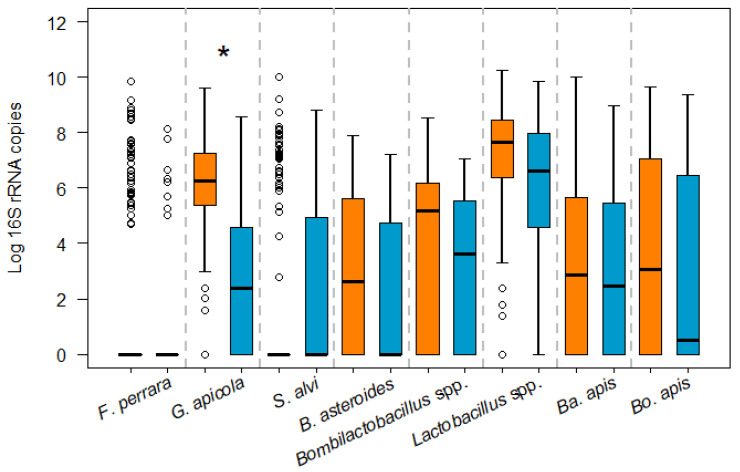
Box-plots showing the absolute abundance of the *16S rRNA* genes for the different gut bacteria analysed in *N. ceranae*-infected (orange) and non-infected bees (blue). The line represents the median, while the box represents 50% of the observations and the whiskers reach the interquartile range of 1.5×. When the distribution of samples does not enable boxes to be established, the bacteria are represented by circles. For pairwise comparisons, the Games–Howell post hoc test was used: * *p* < 0.05.

Regarding the absolute abundance of each of the bacterial species per bee abdomen, only *G. apicola* was found at a significantly higher load in infected bees (Figure 2; GH, *p* = 0.0001). For the other bacterial species, although their abundance was higher in infected bees, except for *S. alvi*, the differences between the infected and non-infected bees were not significant.

### 3.2. Bacterial Loads in Infected and Non-Infected Honey Bees of the Same Age

**Figure 3:**
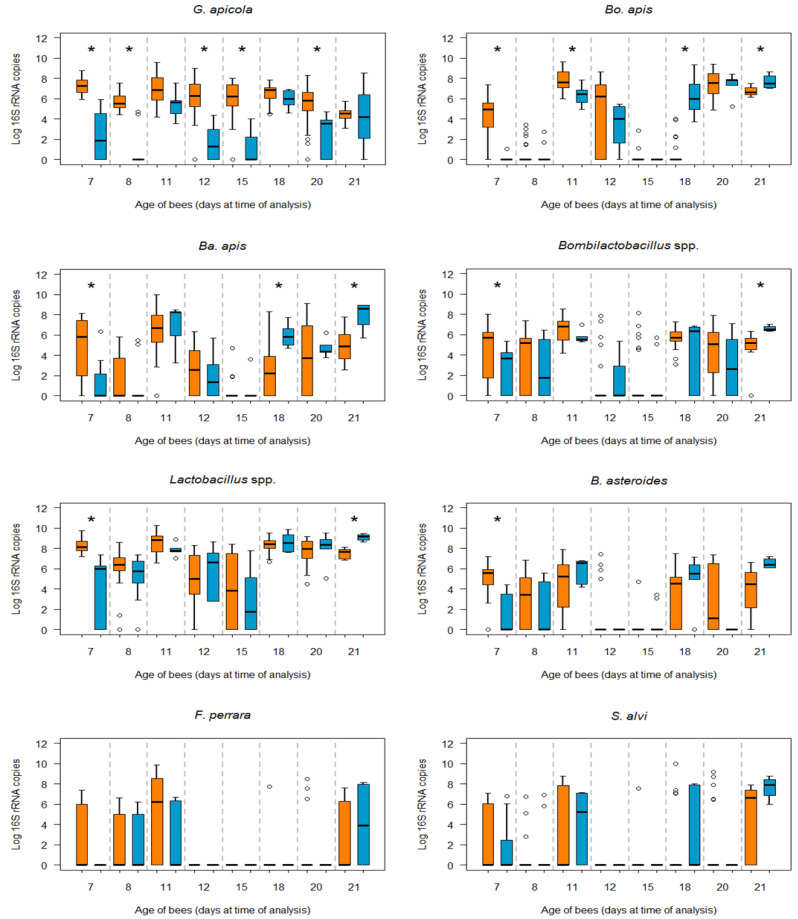
Box-plots showing the absolute copy number abundance of the *16S rRNA* gene for the different gut bacteria analysed in *N. ceranae*-infected (orange) and non-infected (blue) bees at each age, from the highest to lowest differences. The line represents the median, while the box represents 50% of the observations and the whiskers reach the interquartile range of 1.5×. When the distribution of samples does not enable boxes to be established, the bacteria are represented by circles. The Mann–Whitney test was used for pairwise comparisons: * *p* < 0.05.

When the bacterial loads were compared between infected and non-infected bees of the same age, additional differences were observed (Figure 3). Due to the lack of amplification of *Am*-*COI* (see above), the total number of honey bees analysed in each age cohort is shown in Table 3. *G. apicola* was the one species for which significantly higher bacterial loads were detected in infected bees at almost all ages (7, 8, 12, 15, and 20 days p.e.) studied relative to non-infected bees (MW, *p* = 0.0001; MW, *p* = 0.0001; MW, *p* = 0.001; MW, *p* = 0.0001; MW, *p* = 0.006, respectively).

The bacterial load of *Bo. apis* was significantly higher at 7 (MW, *p* = 0.007) and 11 days p.e. in infected bees (MW, *p* = 0.01), yet from 18 days p.e., the *Bo. apis* load was significantly higher in non-infected bees of this age (MW, *p* = 0.0001) and at 21 days p.e. (MW, *p* = 0.007). The same occurred for *Ba. apis*, which was significantly more abundant at 7 days p.e. in infected bees (MW, *p* = 0.02) and in non-infected bees at 18 and 21 days p.e. (MW, *p* = 0.001; MW, *p* = 0.02, respectively). The same trend was evident for the *Bombilactobacillus* spp. and *Lactobacillus* spp., with significantly higher bacterial loads in the younger infected (MW, *p* = 0.018; MW, *p* = 0.0001, respectively) and older non-infected bees (MW, *p* = 0.01; MW, *p* = 0.006, respectively). No significant differences in bacterial loads were observed for *F. perrara* and *S. alvi* between infected and non-infected bees at any age.

**Figure 4:**
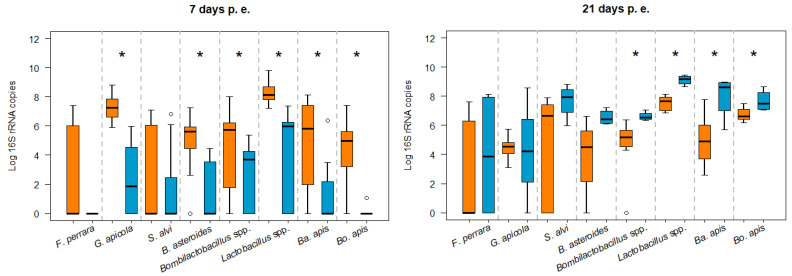
Box-plots showing the absolute abundance of *16S rRNA* gene copy number of the different gut bacteria analysed in 7-day-old (**left**) and 21-day-old (**right**) *N. ceranae*-infected (orange) and non-infected bees (blue). The line represents the median, while the box represents 50% of the observations and the whiskers reach the interquartile range of 1.5×. When the distribution of samples does not enable boxes to be established, the bacteria are represented by circles: * *p* < 0.05.

When we show all of the bees grouped by age, newly emerged infected bees (7 days old at the time of analysis) had higher bacterial loads than non-infected bees, with significant differences for *G. apicola* (MW, *p* = 0.0001), *B. asteroides* (MW, *p* = 0.0001), *Bombilactobacillus* spp. (MW, *p* = 0.018), *Lactobacillus* spp. (*p* = 0.0001), *Ba. apis* (MW, *p* = 0.003), and *Bo. apis* (MW, *p* = 0.0001; Figure 4).

In addition, the oldest non-infected bees in the study (21 days old at the time of analysis) had significantly higher loads of *Ba. apis* (MW, *p* = 0.02), *Bo. apis* (MW, *p* = 0.007), *Bombilactobacillus* spp., and *Lactobacillus* spp. than infected bees of the same age (Figure 4). However, it should be highlighted that the 21-day-old bees in the group of non-infected bees had the least number of bees available (n = 4). Regarding the rest of the ages (8–20 days p.e.), apart from *G. apicola* that was already seen to be the bacteria most significantly influenced by infection, the bacterial load between infected and non-infected bees was only significantly different for *Bo. apis* at 11 and 18 days p.e. (MW, *p* = 0.01; MW, *p* = 0.0001, respectively) and *Ba. apis* at 18 days p.e. (MW, *p* = 0.001). However, while there were no significant differences for the other days, the mean bacterial load in the *N. ceranae*-infected bees was higher than in the non-infected bees, mainly on days 8 and 11 p.e. (Figure S1).

## 4. Discussion

The main objective of this work was to determine whether experimental infection with *N. ceranae* and the age at which the bees are infected influence the main taxa that constitute the honey bee gut microbiota. As such, we studied the main bacterial taxa that represent 95–99% of the honey bee gut bacteriome, showing that of all species tested, *G. apicola* was the species most significantly affected by microsporidium infection, both in terms of presence and bacterial load. This positive association between *G. apicola* and experimental *N. ceranae* infection seems to be very common, as it was found by other authors [56,58,62,63,64] in trials conducted under very different conditions, addressing infection at different ages, different honey bee subspecies, distinct spore doses, gut regions examined, diets fed, analytical methods, or through the analysis of individual bees or pooled samples. However, others failed to find any association between *N. ceranae* infection and this bacterium or with any other bacteria [65], while other studies found associations with other bacteria like those of the genera *Lactobacillus*, *Bifidobacterium*, *Snodgrassella*, or *Bartonella* [61,62,66,67,68,69], which did not appear to be modified here. Most of these studies used very young bees (24–48 h p.e.) and a few slightly older bees (5 d p.e.); however, no study approached the effect of age on the relationship between microbiota and infection, and even this factor is usually not taken into account when comparing results between studies. Nonetheless, our work demonstrates that it is important to take this factor into account, as the results vary depending on the age at which infection occurs. 

To assess the impact of the age at which bees are infected on the gut bacteriome, we performed a comparative analysis of infected and non-infected bees of the same ages. Significant differences between infected and non-infected bees were evident at two key time points, coinciding with the youngest and oldest bees in the trial. Thus, the bees infected just after emergence (7 days old at the time of analysis) had significantly higher bacterial loads for all of the taxa tested, except for *F. perrara* and *S. alvi*, whereas there was a higher abundance of *Bombilactobacillus* spp., *Lactobacillus* spp., *Ba. apis*, and *Bo. apis* in non-infected 21-day-old bees. In the intermediate period, between 8 and 20 days p.e., only occasional differences in bacterial load were observed for any species between infected and non-infected bees, except for *G. apicola*, which was more abundant in *N. ceranae*-infected bees at most ages studied.

It is worth noting that in the work that led to the present research [54], all infected bees were microsporidium-positive; however, 7- and 8-day-old bees (infected at 0 and 1 day p.e.) were much more susceptible to *N. ceranae* infection. This may be because newly emerged bees do not have well-developed peritrophic membranes [68] and the absence of this physical barrier may favour the infection of midgut epithelial cells. Although there were no significant differences in the 8-day-old bees studied here, this trend was similar to that on the previous day. The same was true for bees between 18 and 20 days old in which there were no significant differences, but the tendency towards a higher load of some bacteria for non-infected bees was similar to that on day 21, which was significant. Hence, *N. ceranae* infection apparently alters gut bacteria (in one sense or another depending on age of infeccion), possibly due to damage to the midgut epithelium. Studying a higher sampling size than that used here (mainly for non-infected bees) may produce more robust results and a clearer differentiation.

In this study, *G. apicola* was consistently the species most strongly influenced by *Nosema* infection. Its increased prevalence and abundance in the gut has previously been associated with gut dysbiosis and host deficiencies [16,69,70,71]. The increase in this species and of other non-core bacteria appears to displace the establishment of other core gut bacteria like *S. alvi* [12,16], reducing the protective function of the biofilm that these two species form in the ileum, which, in turn, has been strongly associated with poor host development and early mortality. In fact, *S. alvi* appears at a low frequency in our assay, consistent with the higher prevalence and load of *G. apicola*. Therefore, the increase in this species could be a marker of gut dysbiosis, as suggested previously [16].

On the other hand, the lesions produced by *N. ceranae* in the midgut of bees 7 days after infection (such as the bees study here that were sacrificed 7 days after infection regardless of their age), with signs of degeneration in most epithelial cells [39,72], could affect midgut function and negatively affect food digestion and nutrient absorption [73,74,75]. Therefore, the sucrose from the food, which is hydrolysed into glucose and fructose by a sucrase secreted by the hypopharyngeal glands [76], would not be correctly absorbed by the injured midgut epithelium, and would pass into the ileum and rectum. This would also be the case with amino acids and vitamins, where they could then be utilised by gut bacteria as a substrate [77,78], potentially producing a further imbalance in the gut microbiota. Moreover, several other effects of *N. ceranae* infection already described could contribute to the altered digestive tract homeostasis, such as those affecting the regulation of antioxidant systems, reducing reactive oxygen species (ROS) production [39,79,80], or enhancing immunosuppression by inhibiting the production of antimicrobial peptides (AMPs) [42,65,81]. Indeed, microsporidia infection in other insects induces acidification and increased ROS in the hindgut, reducing bacterial diversity and affecting the structure of the gut bacterial community [82]. Therefore, by disrupting the gut ecosystem, possibly through changes in AMPs, ROS, or pH, *N. ceranae* could influence bacterial communities. Here, all of these imbalances produce either an increase or a decrease in bacterial species.

In this way, the non-absorption of sugars in the midgut and their passage to the ileum could explain why infected bees have more abundant bacteria. In addition, *G. apicola* is one of the first bacteria to colonise the ileum, and it can utilise glucose and fructose simultaneously [83], possessing complete metabolic pathways for the utilisation of all amino acids [84]. As a result, this species will benefit from the influx of these nutrients, explaining why it is one of the bacterial species most influenced by microsporidium infection in this study.

Our work shows that the age at which bees become infected with *N. ceranae* has a clear influence on the gut bacteriome. Thus, newly emerged bees receive the microsporidium and the gut microbiota at virtually the same time, or within a few hours of each other. By contrast, all of the other bees already had certain bacteria in their digestive tract at the time of infection, and the stability of these bacteria in the gut is age-dependent. Indeed, bees infected at 24 h p.e. still do not have a well-established microbiota, whereas those infected at 14 days p.e. have a more established microbiota [14,15]. Nevertheless, it is noteworthy that these results were obtained under laboratory conditions and, evidently, they differ from field conditions. In fact, honey bees become naturally infected by *N. ceranae* between 4 and 5 days p.e. inside the colony [50], and in the studies carried out under field conditions, there is less disparity in the gut microbiota of bees relative to infection. The effects reported range from no difference between infected and non-infected bees [85] to a higher abundance of bifidobacteria in *N. ceranae*-infected bees [57] or of *Bartonella* in honey bees with high infection levels [86]. The bees in these studies had developed part of their life inside the colony, allowing them to naturally acquire their characteristic gut microbiota. From the moment a new worker emerges, there are multiple ways in which they acquire and develop their microbiota, such as contact with elements in the hive, interactions with other older bees, and the consumption of natural foods, among others [21], which, of course, are limited in laboratory assays. Therefore, it is plausible that the microbiota has already begun to be established when the bees are naturally infected and, therefore, susceptibility to infection may differ from that of bees experimentally infected in the laboratory and kept in the lab throughout the assay.

Nevertheless, most of the bees in our study possessed the main characteristic bacterial taxa in their guts despite not having developed their natural life cycle inside the hive, but, rather, having acquired these bacteria through contact with the brood frame in the first 24 h after emergence, with *S. alvi* being a notable exception. Special care was taken to ensure that the *N. ceranae* spore inoculum did not bias the study, providing the bees of the infected group with gut bacteria. In fact, qPCR analysis of the spore inoculum indicated that it was free of the bacterial species studied, such that only *N. ceranae* spores were administered to the bees. In other words, the bacteria detected in this study could only have been acquired through contact with the brood frame. These results indicate that natural emergence and brief exposure to the brood frame is sufficient to acquire microbiomes very similar to those found in the hive environment, as described previously [21,87].

## 5. Conclusions

This study revealed that *N. ceranae* infection, irrespective of age, seems to have a notable impact on the composition of the honey bee gut microbiota, with *G. apicola* being the bacterium most profoundly affected both in terms of presence and bacterial load. Moreover, the age of infection was confirmed to be an important factor to be considered in these studies, as the age at which the bees were infected with microsporidium influenced the abundance of gut bacterial species. Younger infected bees have a higher abundance of virtually all of the bacterial species analysed, while the same occurred for some bacteria in older non-infected bees.

Thus, our results clearly show that the microsporidia *N. ceranae* can alter the gut microbiota of honey bees under laboratory conditions. It would therefore be interesting to further study the interactions between the microsporidian and the gut microbiota with the aim of finding a possible solution to the negative effects of this widespread pathogen on honey bees.

## Figures and Tables

**Table 1:** Number of infected and non-infected bees analysed in each age cohort.

Age of Infection (Days p.e.)	0	1	4	5	8	11	13	14	Total
Age of Analysis (Days p.e.)	7	8	11	12	15	18	20	21	
Infected bees (N)	30	30	30	30	28	30	30	7	215
Non-infected bees (N)	11	10	6	6	10	6	6	6	61

**Table 2:** Primers and probe to detect and quantify the *16S rRNA* gene of *Bo. apis* (Alpha 2.2): F, forward; R, reverse; P, probe. The LNAs (+) added to the probe are indicated.

Primers	Sequence (5′-3′)	Size
ALPHA 2.2-F	CCGAGAGAGGGTTGTGGAATT	67 pb
ALPHA 2.2-R	AGATATTGGGAAGAACACCG	
ALPHA 2.2-P	6FAM-TGTAGA+GG+T+GAA+AT+TC-BHQ1	

**Table 3:** Number of honey bees included in the statistical analysis.

Age of Infection (Days p.e.)	0	1	4	5	8	11	13	14	Total
Age of Analysis (Days p.e.)	7	8	11	12	15	18	20	21	
Infected bees (N)	28	30	27	27	27	30	30	7	206
Non-infected bees (N)	11	10	5	6	10	6	5	4	57

## Data Availability

Data are contained within the article and Supplementary Materials.

## Supplementary Materials

The following supporting information can be downloaded at: https://www.mdpi.com/article/10.3390/microorganisms12040635/s1, Table S1: GenBank (NCBI) accession numbers for primer and probe design for the detection and quantification of *Bombella apis*.; Table S2: The gBlock^®^ Gene Fragment ordered for the bacterial *16S rRNA* gene and the *Apis mellifera COI* gene. The primers (red) and probes (blue) annealing regions are indicated in bold and underlined; Table S3: Primers used in this study for the bacterial *16S rRNA* gene and the *Apis mellifera COI* gene and standard curve characteristics. LOD refers to the limit of detection of primer sets, expressed as the lowest number of DNA copies detected when standard curves were performed. F: Forward; R: Reverse; P: probes; Tm: Melting temperature; Figure S1: Box-plots showing the absolute abundance of *16S rRNA* gene copy number of the different gut bacteria analysed in *N. ceranae* infected and non-infected bees at each age. The line represents the median, while the box represents 50% of the observations and the whiskers reach the interquartile range of 1.5×. Cases where the distribution of samples does not allow for boxing are represented by circles. For pairwise comparisons, the Mann–Whitney test was used. * *p* < 0.05.
