# Supplementary material for: Influence of Age of Infection on the Gut Microbiota in Worker Honey Bees (Apis mellifera iberiensis) Experimentally Infected with Nosema ceranae
Source: Microorganisms. 2024 Mar 22;12(4):635. doi: 10.3390/microorganisms12040635 (PMC11051791; doi:10.3390/microorganisms12040635)

Figure S1. Box-plots showing the absolute abundance of *16S rRNA* gene copy number of the different gut bacteria analysed in *N. ceranae* infected and non-infected bees at each age. The line represents the median, while the box represents 50% of the observations and the whiskers reach the interquartile range of 1.5x. Cases where the distribution of samples does not allow for boxing are represented by circles. For pairwise comparisons, the Mann-Whitney test was used. *p<0.05.

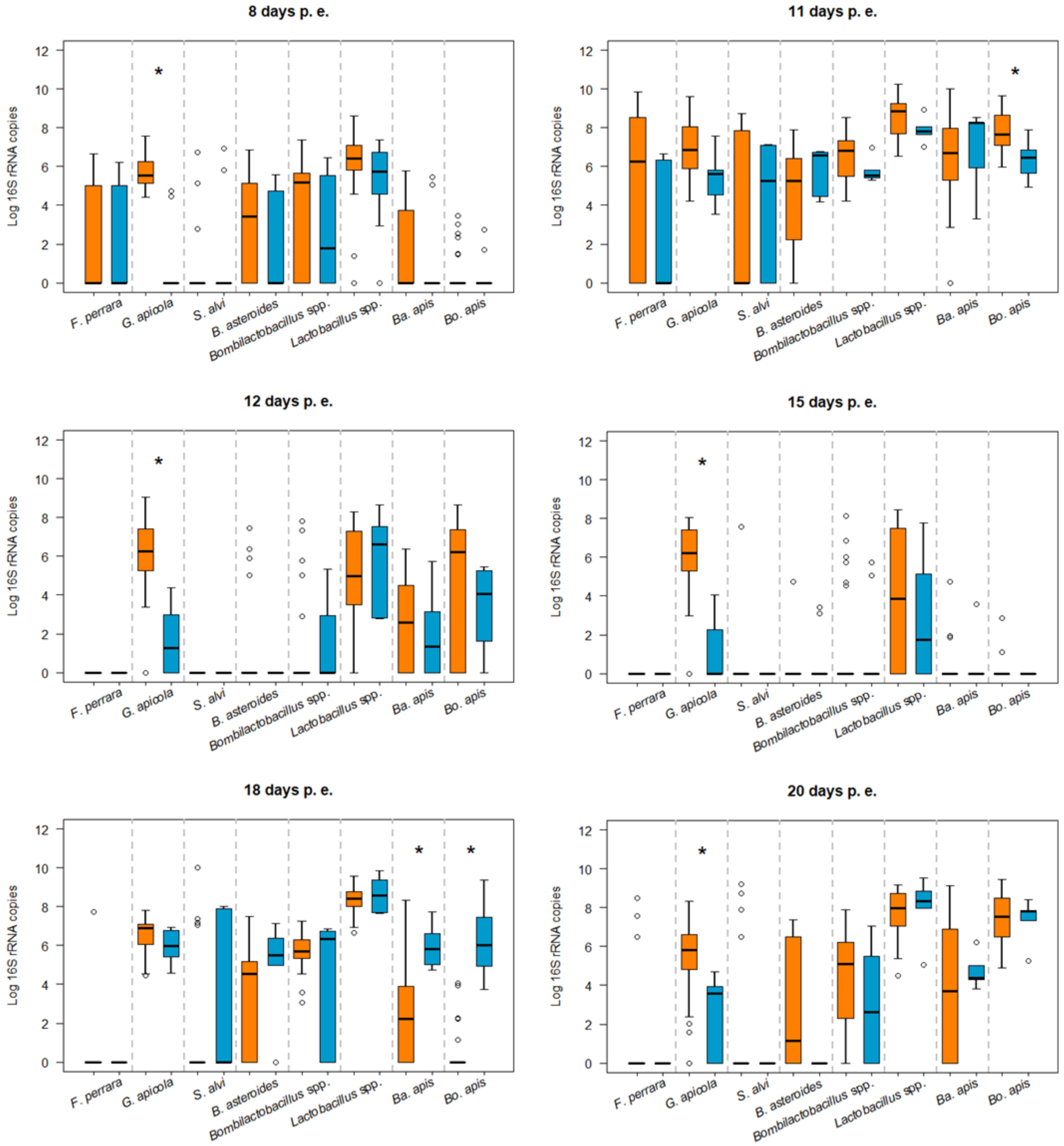

Supplement: Supplementary file 1 [file microorganisms-12-00635-s001.zip › Figure S1.pdf]
