# Supplementary material for: Influence of Age of Infection on the Gut Microbiota in Worker Honey Bees (Apis mellifera iberiensis) Experimentally Infected with Nosema ceranae
Source: Microorganisms. 2024 Mar 22;12(4):635. doi: 10.3390/microorganisms12040635 (PMC11051791; doi:10.3390/microorganisms12040635)
Supplement: Supplementary file 1 [file microorganisms-12-00635-s001.zip › Table S1.pdf]

Table S1. GenBank (NCBI) accession numbers for primer and probe design for the detection and quantification of *Bombella apis*.

Species	Access Number
P. apium	NZ_CP020554.1; NR_133042.1; KM014167.1; KM014144.1; MH205731.1; KT598292.1; NZ_LMYI01000023.1; NZ_LMYJ01000028.1; NZ_LMYH01000022.1; MEJG01000010.1.
S. floricola	KJ638086.1
Bo. apis	NR_157653.1; KU534110.1; MN582688.1; NZ_JAGJTM010000006.1; NZ_JAGJTM010000001.1; NZ_JAGJTM010000002.1; NZ_JADAQV010000006.1; NZ_WHNS01000003.1; NZ_JAGJTM010000005.1; NZ_WHNS01000007.1; NZ_WHNS01000005.1; NZ_WHNS01000002.1; CP046394.1; MT787561.1; MT787560.1.
