# Supplementary material for: Influence of Age of Infection on the Gut Microbiota in Worker Honey Bees (Apis mellifera iberiensis) Experimentally Infected with Nosema ceranae
Source: Microorganisms. 2024 Mar 22;12(4):635. doi: 10.3390/microorganisms12040635 (PMC11051791; doi:10.3390/microorganisms12040635)
Supplement: Supplementary file 1 [file microorganisms-12-00635-s001.zip › Table S2.pdf]

Table S2. The gBlock® Gene Fragment ordered for the bacterial *16S rRNA* gene and the *Apis mellifera COI* gene. The primers (red) and the probes (blue) annealing regions are indicated in bold and underlined.

Bacteria	Sequence gBlock® Gene Fragment (5′ to 3′)	Size (pb)
Frischella perrara (*16S rRNA*, NR_118490.1)	AGATTAGCTAGTTGGTGGGGTAAAGGCTCACCAAG<br>GCGACGATCTCTAGCTGGTCTGAGAGGATGACCAG<br>CCACACTGGAACTGAGACACGGTCCAGACTCCTAC<br>GGGAGGCAGCAGTGGGGAATATTGCACAATGGGG<br>GAAACCCTGATGCAGCCATGCCGCGTGTATGAAGA<br>AGGCCTTAGGGTTGTAAAGTACTTTCGGTGATGA**G**<br>**GAAGTTATGTGTGGGATAAGC**ACATATAATTGAC<br>GTTAGTTACAGAAGAAGCACCGGCTAACTCCGTGC<br>CAGCAGCCGCGGTAATACGGAGGGTGCGAGCGTTA<br>ATCGGAATGACTGGGCGTAAAGGGCATGTAGGCGG<br>ATGATTAAGTTAGGTGTGAAAGCCT**CGGGCTCAAC**<br>**CTGAGAATAG**CATTTAAAACTGGTGATCTGGAGTA<br>CTGTAGAGGGAGGTAGAATTCCACGTGTAGCGGTG<br>AAATGCGTAGAGATGTGGAGGAATACCGGTGGCGA<br>AG	490
Gilliamella apicola (*16S rRNA*, NR_121727.1)	GCACAAGCGGTGGAGCATGTGGTTTAATTCGATGC<br>AACGCGAAGAACCTTACCTGGTCTTGACATCCACA<br>GAATCTTGCAGAGATGCGGGAGTGCCTTCGGGAAC<br>TGTGAGACAGGTGCTGCATGGCTGTCGTCAGCTCGT<br>GTTGTGAAATGTTGGGTTAAGTCCCGCAACGAGCG<br>CAACCCTTATC**CTTTGTTGCCATCGGTTAGGCC**GG<br>GAACTCAAAGGAGACTGCCGTTGATAAAGCGGAGG<br>AAGGTGGGGACGACGTCAAGTCATCATGGCCCTTA<br>CGACCAGGGCTACACACGTGCTACAATGGCGTATA<br>CAAAGGGAGGCGA**CCTCGCGAGAGCAAGCGG**AC<br>CTCATAAAGTACGTCTAAGTCCGGATTGGAGTCTGC<br>AACTCGACTCCATGAAGTCGGAATCGCTAGTAATC<br>GTGAATCAGAATGTCACGGTGAATACGTTCCCGGG<br>CCTTGTACACACCGCCCGTCACACCATGGGAG	487
Snodgrassella alvi (*16S rRNA*, NR_122055.1)	AACGATGACAATTAGCTGTTGGGACACTAGATGTCT<br>TAGTAGCGAAGCTAACGCGTGAAATTGTCCGCCTG<br>GGGAGTACGGTCGCAAGATTAAAACTCAAAGGAAT<br>TGACGGGGACCCGCACAAGCGGTGGATGATGTGGA<br>TTAATTCGATGCAACGCGAAGAACCTTACCTGGTCT<br>TGACATGTACGGAATCT**CTTAGAGATAGGAGAGT**<br>**GCCTT**CGGGAACCGTAACACAGGTGCTGCATGGCT<br>GTCGTCAGCTCGTGTCGTGAGATGTTGGGTTAAGTC<br>CCGCAACGAGCGCAACCC**TTGTCATTAGTTGCCAT**<br>**CATTAAGTT**GGGCACTCTAATGAGACTGCCGGTGA<br>CAAACCGGAGGAAGGTGGGGATGACGTCAAGTCCT<br>CATGGCCCTTATGACCAGGGCTTCACACGTCATACA<br>ATGGTCGGTACAGAGGGTAGCGAAGCCGCGAGGTG<br>AAGCCAATCTCAGAAAGCCGATCGTAGTCCG	489
Bifidobacterium asteroides (*16S rRNA*, NR_044154.1)	AACAC**ATGCAAGTCGAACGGGATCC**GGGCAGCTT<br>GCTGCCTGGTGAGAGTGGCGAACGGGTGAGTAATG<br>CGTGACCAACCTGCCCCATGCTTCGGAATAGCTCCT<br>GGAAACGGGTGGTAATGCCGGATGCTCCGCACCGT	500

	CGCATGATGGTGTGGGAAA**GGGTTTACCGGCATG**<br>**GGATG**GGGTCGCGTCCTATCAGCTTGTTGGCGGGG<br>TGATGGCCTGCCAAGGCTTCGACGGGTAGCCGGCC<br>TGAGAGGGCGACCGGCCACATTGGGACTGAGATAC<br>GGCCCAGACTCCTACGGGAGGCAGCAGTGGGGAAT<br>ATTGCACAATGGGCGCAAGCCTGATGCAGCGACGC<br>CGCGTGCGGGATGACGGCCTTCGGGTTGTAAACCG<br>CTTTTGATTGGGAGCAAGCGAGAGTGAGTGTACCTT<br>TCGAATAAGCACCGGCTAACTACGTGCCAGCAGCC<br>GCGGTAATACGTAGGGTGCAAGCGTTATCCGGATTT<br>ATTGGGCGT	
Bombilactobacillus mellis (*16S rRNA*, NR_126249.1)	TGCA**AGTCGAGCGCGGGAAGTCA**GGGAAGCCTTC<br>GGGTGGAACTGGTGGAACGAGCGGCGGATGGGTGA<br>GTAACACGTAGGTAACCTGCCCTAAAGCGGGGGAT<br>ACCATCTGGAAACAGGTGCTAATACCGCATAACCC<br>CAGCAGTCACATG**AGTGCTGGTTGAAAGACGGCT**<br>TCGGCTGTCACTTTAGGATGGACCTGCGGCGTATTA<br>GCTAGTTGGTGGAGTAACGGTTCACCAAGGCGATG<br>ATACGTAGCCGACCTGAGAGGGTAATCGGCCACAT<br>TGGGACTGAGACACGGCCCAAACTCCTACGGGAGG<br>CAGCAGTAGGGAATCTTCCACAATGGACGCAAGTC<br>TGATGGAGCAACGCCGCGTGGATGAAGAAGGTCTT<br>CGGATCGTAAAATCCTGTTGTTGAAGAAGAACGGT<br>TGTGAGAGTAACTGCTCATAGCGTGACGGTAATCA<br>ACCAGAAAGTCACGGCTAACTACGTGCCAGCAGCC<br>GC	491
Lactobacillus helsingborgensis (*16S rRNA*, NR_126253.1)	TGCAAGTCGAGCGAGCAATTTTGACGGAATACTTC<br>GGTAGGAAGTCAGAAGCGCGAGCGGCGGATGGGT<br>GAGTAACACGTGG**GCAACCTGCCCTTTAGCTTG**GG<br>ATACCACTTGGAAACAGGTGCTAATACCAAATAAG<br>CAGCACGAGCGCATGCTCGAGCTGAGAAAGGCGG<br>CTTTCGA**GCTGTCACTAAAGGATGGGC**CCGCGGT<br>GCATTAGCTAGTTGGTAAGGTAACGGCTTACCAAG<br>GCGATGATGCATAGCCGAGTTGAGAGACTGACCGG<br>CCACATTGGGACTGAGACACGGCCCAAACTCCTAC<br>GGGAGGCAGCAGTAGGGAATCTTCCACAATGGACG<br>CAAGTCTGATGGAGCAACGCCGCGTGAGTGAAGAA<br>GGTTTTCGGATCGTAAAGCTCTGTTGTTGGTGAAGA<br>AGGACAAGGGTAGTAACTGATTCTTGTTTGACGGTA<br>ATCAACCAGAAAGTCACGGCTAACTACGTGCCAGC<br>AG	491
Bartonella apis (*16S rRNA*, NR_126253.1)	AAGCCAAAATCAAATTTTCAACTTGAGAGTTTGATC<br>CTGGCTCAGAACGAACGCTGGCGGCAGGCTTAACA<br>CATGCAAGTCGAACGCACTTTTCGGAGTGAGTGGC<br>AGACGGGTGAGTAACGC**GTGGGAATCTACCTATT**<br>**TCTACG**GAATAACGCAGAGAAATTTGTGCTAATAC<br>CGTATACGTCCTTCGGGAGAAAGATTTATCG**GAGA**<br>**TAGATGAGCCCGCGTT**GGATTAGCTAGTTGGTGAG<br>GTAATGGCCCACCAAGGCGACGATCCATAGCTGGT<br>CTGAGAGGATGACCAGCCACATTGGGACTGAGACA<br>CGGCCCAGACTCCTACGGGAGGCAGCAGTGGGGAA	471

	TATTGGACAATGGGCGCAAGCCTGATCCAGCCATG<br>CCGCGTGAGTGATGAAGGCCCTAGGGTTGTAAAGC<br>TCTTTCACCGGTGAAGATAATGACGGTAACCGGAG<br>AAGAAGCCCCGGCTAA	
Bombella apis (*16S rRNA*, NR_147774.1)	AAGATGATGACGGTACCTAGAGAAGAAGCCCCGG<br>CTAACTTCGTGCCAGCAGCCGCGGTAATACGAAGG<br>GGGCTAGCGTTGCTCGGAATGACTGGGCGTAAAGG<br>GCGCGTAGGCTGTCTGTACAGTCAGATGTGAAATCC<br>CCGGGCTTAACCTGGGAACTGCATTTGATACGTGCA<br>GACTAGAGT**CCGAGAGAGGGTTGTGGAATT**CCCA<br>G**TGTAGAGGTGAAATTC**GT**AGATATTGGGAAGA**<br>**ACACCG**GTTGCGAAGGCGGCAACCTGGCTCGGAAC<br>TGACGCTGAGGCGCGAAAGCGTGGGGAGCGAACA<br>GGATTAGATACCCTGGTAGTCCACGCTGTAAACGAT<br>GTGTGCTGGATGTTGGGTGATTTTATCATTCAGTGTC<br>GGAGCTAACGCGTTAAGCACACCGCCTGGGGAGTA<br>CGGCCGCAAGGTTGAAACTCAAAGGAATTGACGGG<br>GGCCCGCACAAGCGGTGGAGCATGTGGTTTAATTC	490
Apis mellifera (*citocrome oxidase I*, OM203346.1)	GTTATACCATTTTTAATTGGAGGATTTGGAAATTGG<br>CTTATTCCTTTAATACTAGGATCACCTGATATAGCA<br>TTCCCCCGAATAAATAATATTAGATTTTGATTACTTC<br>CTCCCTCATTATTTATACTTTTATTAAGAAATTTATTT<br>TA**TCCAAGACCAGGAACTGGAT**GAACAGTATATC<br>CACCATTATCAGCATATTTATATCATTCTTCACCTTC<br>AGTAGATTTTGCAATT**TTTTCTCTTCATATATCAGG**<br>**AATTTCC**TCAATTATAGGATCATTAAACTTAATAGT<br>TACAATTATAATAATAAAAAATTTTTCTATAAATTA<br>TGACCAAATTTCATTATTTCCATGATCAGTTTTTATT<br>ACAGCAATTTTATTAATTATATCATTACCTGTATTAG<br>CTGGAGCAATTACTATACTATTATTTGATCGAAATT<br>TTAATACATCATTTTTCGATCCTATAGGAGGTGGAG<br>ATCCAATTCTTTATCAACATTTA	495
