# Supplementary material for: Influence of Age of Infection on the Gut Microbiota in Worker Honey Bees (Apis mellifera iberiensis) Experimentally Infected with Nosema ceranae
Source: Microorganisms. 2024 Mar 22;12(4):635. doi: 10.3390/microorganisms12040635 (PMC11051791; doi:10.3390/microorganisms12040635)
Supplement: Supplementary file 1 [file microorganisms-12-00635-s001.zip › Table S3.pdf]

Table S3. Primers used in this study for the bacterial *16S rRNA* gene and the *Apis mellifera COI* gene and standard curve characteristics. LOD refers to the limit of detection of primer sets, expressed as the lowest number of DNA copies detected when standard curves were performed. F: Forward; R: Reverse; P: Probes; Tm: Melting temperature.

Target	Sequence (5′ to 3′)	Amplicon size (pb)	Standard curves						References
			Tm (°C)	Efficiency (%)	Error	Slope	Intercept	LOD Cp (copies)	
Frichella perrara	F: GGAAGTTATGTGTGGGATAAGC<br>R: CTATTCTCAGGTTGAGCCCG	185	84.4	1,937 (96.85)	0,0514	-3,482	7,629	35,75 (10^2)	[23]
Gilliamella apicola	F: CTTTGTTGCCATCGGTTAGGCC<br>R: CCGCTTGCTCTCGCGAGG	160	83.6	2,032 (101.6)	0,0191	-3,246	5,727	39,06 (10^2)	[23]
Snodgrassella alvi	F: CTTAGAGATAGGAGAGTGCCTT<br>R: AACTTAATGATGGCAACTAATGACAA	132	84.6	1,926 (96.3)	0,0176	-3,514	11,08	38,43 (10^2)	[23]
Bifidobacterium asteroides	F: ATGCAAGTCGAACGGGATCC<br>R: CATCCCATGCCGGTAAACCC	174	85.8	2,013 (100.6)	0,0360	-3,290	6,54	36,94 (10)	[23]
Bombilactobacillus spp.	F: AGTCGAGCGCGGGAAGTCA<br>R: AGCCGTCTTTCAACCAGCACT	169	84.5	2,043 (102.1)	0,0228	-3,224	4,403	36,4 (10)	[23]
Lactobacillus spp.	F: GCAACCTGCCCTWTAGCTTG<br>R: GCCCATCCTKTAGTGACAGC	118	81.3	1,964 (98.2)	0,0214	-3,412	5,242	39,52 (10)	[23]
Bartonella apis	F: GTGGGAATCTACCTATTTCTACG<br>R: AACGCGGGCTCATCTATCTC	103	76.6	2,016 (100.8)	0,0255	-3,285	5,333	37,78 (10)	[23]
Bombella apis	F: CCGAGAGAGGGTTGTGGAATT<br>R: AGATATTGGGAAGAACACCG<br>P: TGTAGA+GG+T+GAA+AT+TC	65	-	2,057 (102.8)	0,0185	-3,193	5,775	38,5 (2)	This study
Apis mellifera	F: TCCAAGACCAGGAACTGGAT<br>R: TTTTCTCTTCATATATCAGGAATTTCC	112	75.2	1,953 (97.7)	0,0601	-3,439	12,71	34,87 (10^5)	[60]
